# Supplementary material for: Prevalence of Helicobacter pylori, Salmonella typhi, Plasmodium falciparum, and Toxoplasma gondii infections and levels of liver function markers among Hepatitis B Virus infected Ghanaians: A cross-sectional study in the Greater Accra Region
Source: PLOS Glob Public Health. 2025 Sep 5;5(9):e0005132. doi: 10.1371/journal.pgph.0005132 (PMC12412962; doi:10.1371/journal.pgph.0005132)
Supplement: S1 File — (DOCX) [file pgph.0005132.s001.docx]

Blood sample collection was done at the Mamprobi Polyclinic, Ussher Polyclinic, and Kaneshie Polyclinic

151 patients who tested positive to the HBsAg were included

120 samples were further screened and grouped into HBV-only, HBV-H.pylori, HBV-S.typhi, HBV-P.falciparum, and HBV-T. gondii groups

Patients who tested negative to the HBsAg were excluded

- Among the HBV -HBsAg positive patients, 31 were excluded, including:
- Those who tested positive for either HIV, HCV, or HAV and had coinfections with 2 or more of *H. pylori, S. typhi, P. falciparum and T. gondii,*

- Thos who had taken antimicrobials against any of the coinfected pathogens in ≤3 months

**Or**

- Were Pregnant women
