# Supplementary material for: Prevalence of Helicobacter pylori, Salmonella typhi, Plasmodium falciparum, and Toxoplasma gondii infections and levels of liver function markers among Hepatitis B Virus infected Ghanaians: A cross-sectional study in the Greater Accra Region
Source: PLOS Glob Public Health. 2025 Sep 5;5(9):e0005132. doi: 10.1371/journal.pgph.0005132 (PMC12412962; doi:10.1371/journal.pgph.0005132)
Supplement: S2 File — (DOC) [file pgph.0005132.s002.doc]

**STROBE checklist.**

**Title: Prevalence of Helicobacter pylori, Salmonella typhi, Plasmodium falciparum, and Toxoplasma gondii infections and levels of liver function markers among Hepatitis B Virus infected Ghanaians: A cross-sectional study in the Greater Accra Region**

|  | | Item No | Recommendation | Page(s) |
| --- | --- | --- | --- | --- |
| **Title and abstract** | | 1 | (*a*) Indicate the study’s design with a commonly used term in the title or the abstract  *Cross-sectional as stated in the Title, Abstract on pages 1 and 2, and Design/ Methods on page 5.* | 1,2 |
|  |  |  | (*b*) Provide in the abstract an informative and balanced summary of what was done and what was found  *Provided in Abstract on pages 1/2.* | 2 |
| Introduction | | | |  |
| Background/rationale | | 2 | Explain the scientific background and rationale for the investigation being reported.  *Included in the Introduction on pages 3,4 and 5* | 3,4, 5 |
| Objectives | | 3 | State specific objectives, including any prespecified hypotheses.  *Included in the Introduction on pages 4 and 5* | 4,5 |
| Methods | | | |  |
| Study design | | 4 | Present key elements of study design early in the paper  *Included in the Methods on page 5* | 5 |
| Setting | | 5 | Describe the setting, locations, and relevant dates, including periods of recruitment, exposure, follow-up, and data collection.  *Included in the Methods on pages 5 and 6* | 5,6 |
| Participants | | 6 | (*a*) *Cohort study*—Give the eligibility criteria, and the sources and methods of selection of participants. Describe methods of follow-up  *Case-control study*—Give the eligibility criteria, and the sources and methods of case ascertainment and control selection. Give the rationale for the choice of cases and controls.  *Cross-sectional study*—Give the eligibility criteria, and the sources and methods of selection of participants.  *Included in the Methods on pages 5,6,7 and 8* | 5,6,7,8 |
|  |  |  | (*b*) *Cohort study*—For matched studies, give matching criteria and number of exposed and unexposed  *Case-control study*—For matched studies, give matching criteria and the number of controls per case. |  |
| Variables | | 7 | Clearly define all outcomes, exposures, predictors, potential confounders, and effect modifiers. Give diagnostic criteria, if applicable  *Included in the Methods on pages 6,7 and 8* | 6,7,8 |
| Data sources/ measurement | | 8* | For each variable of interest, give sources of data and details of methods of assessment (measurement). Describe comparability of assessment methods if there is more than one group.  *Included in the Methods on pages 6,7 and 8* | 6,7,8 |
| Bias | | 9 | Describe any efforts to address potential sources of bias. |  |
| Study size | | 10 | Explain how the study size was arrived at.  *Included in the Methods on page 5,6* | 5,6 |
| Quantitative variables | | 11 | Explain how quantitative variables were handled in the analyses. If applicable, describe which groupings were chosen and why.  *Included in the Methods on pages 5,6,7,8* | 5,6,7,8 |
| Statistical methods | | 12 | (*a*) Describe all statistical methods, including those used to control for confounding  *Included in the Methods on page 8* | 8 |
|  |  |  | (*b*) Describe any methods used to examine subgroups and interactions  . |  |
|  |  |  | (*c*) Explain how missing data were addressed |  |
|  |  |  | (*d*) *Cohort study*—If applicable, explain how loss to follow-up was addressed  *Case-control study*—If applicable, explain how matching of cases and controls was addressed.  *Cross-sectional study*—If applicable, describe analytical methods taking account of sampling strategy *Included in the Methods on pages 6,7,8* | 5,6,7,8 |
|  |  |  | (*e*) Describe any sensitivity analyses |  |
| Results | | | |  |
| Participants | 13* | (a) Report numbers of individuals at each stage of study—eg numbers potentially eligible, examined for eligibility, confirmed eligible, included in the study, completing follow-up, and analysed  *Included in the Results on pages 8,9,10* | | 8,9,10 |
|  |  | (b) Give reasons for non-participation at each stage | |  |
|  |  | (c) Consider use of a flow diagram*.* | |  |
| Descriptive data | 14 | (a) Give characteristics of study participants (eg demographic, clinical, social) and information on exposures and potential confounders  Included in the Results on pages 8,9,10 | | 8,9,10,11 |
|  |  | (b) Indicate number of participants with missing data for each variable of interest | |  |
|  |  | (c) *Cohort study*—Summarise follow-up time (eg, average and total amount) | |  |
| Outcome data | 15* | *Cohort study*—Report numbers of outcome events or summary measures over time | |  |
|  |  | *Case-control study—*Report numbers in each exposure category, or summary measures of exposure | |  |
|  |  | *Cross-sectional study—*Report numbers of outcome events or summary measures *Included in the Results on pages 10 -13, and summarized in Tables 1, Table 2a & 2b, and Figure 1.* | | 10,11,12,13  Fig 1, Tables 1,2a &2b |
| Main results | 16 | (*a*) Give unadjusted estimates and, if applicable, confounder-adjusted estimates and their precision (eg, 95% confidence interval). Make clear which confounders were adjusted for and why they were included.  *Not applicable* | |  |
|  |  | (*b*) Report category boundaries when continuous variables were categorized  *Not applicable* | |  |
|  |  | (*c*) If relevant, consider translating estimates of relative risk into absolute risk for a meaningful period. *Not applicable.* | |  |
| Other analyses | 17 | Report other analyses done—eg analyses of subgroups and interactions, and sensitivity analyses.  *Included in the Results on page 10* | | 10,11 |
| Discussion | | | |  |
| Key results | 18 | Summarise key results with reference to study objectives.  *Included in the Discussion on page 13 to 17.* | | 13,14,15,16,17 |
| Limitations | 19 | Discuss limitations of the study, considering sources of potential bias or imprecision. Discuss both direction and magnitude of any potential bias  *Included on page 17 &18* | | 17 &18 |
| Interpretation | 20 | Give a cautious overall interpretation of results considering objectives, limitations, multiplicity of analyses, results from similar studies, and other relevant evidence.  *Included in the Discussion on pages 13 to 17* | | 13,14,15,16,17 |
| Generalisability | 21 | Discuss the generalisability (external validity) of the study results.  *Included in the Discussion on page 13 to 17* | | 13,14,15,16,17 |
| Other information | | | |  |
| Funding | 22 | Give the source of funding and the role of the funders for the present study and, if applicable, for the original study on which the present article is based.  *Provided in the text on page 21.* | | 21 |
