# Supplementary material for: Prevalence of Helicobacter pylori, Salmonella typhi, Plasmodium falciparum, and Toxoplasma gondii infections and levels of liver function markers among Hepatitis B Virus infected Ghanaians: A cross-sectional study in the Greater Accra Region
Source: PLOS Glob Public Health. 2025 Sep 5;5(9):e0005132. doi: 10.1371/journal.pgph.0005132 (PMC12412962; doi:10.1371/journal.pgph.0005132)
Supplement: Attachment — Submitted filename: R1 10 Response to Reviewers.docx [file pgph.0005132.s004.docx]

19^th^ December 2024

Dear Reviewer (s) & Editor,

**Point-by-point response to reviewers**

(**RE**: Prevalence of H. pylori, S. typhi, P. falciparum, and T. gondii infections and levels of liver function markers among HBV infected Ghanaians: A cross-sectional study in the Greater Accra Region) (**PGPH-D-24-02628**)

Thank you for your thorough review, suggestions, and comments. Please find responses:

**Questions/ Comments:**

1. The sample source is not well described. Please provide details for the current descriptions in lines 115 and 116. Is the HBV routine checking in these health facilities? Is there an algorithm to find these patients? Is it something designed just for this study?

**Response**: No, HBV testing is readily available in these facilities, but it is **not** routinely checked, except for Pregnant women, and persons with other forms of STIs willing to have voluntary HBV counselling and testing. All other patients/clients would check their HBV status upon a caretaker’s suspicion of exposure based on examination, signs and symptoms, or a client’s request to know status for vaccination, migration or relationship purposes.

There were (combined from the 3 facilities) records (with contacts) of over 260 people who had previously tested positive for HBV. Some reported in the period of the study, those who consented among this group were included, while others were invited via phone call – again those who reported and consented were included. Also, there were others who had come to the facility for screening, due to any of the previous reasons; they were contacted and those who consented were included. In all, 151 patients who tested positive for the HBsAg were initially included, but 31 persons were further excluded - upon testing positive for either HIV, HCV, or HAV, and or had coinfections with 2 or more of *H. pylori, S. typhi, P. falciparum and T. gondii*; taken antimicrobial in ≤ 3months or Pregnant. This brought the total inclusion to 120 patients/ participants.

Yes, we used a kind of simplified flow chart (**SF1**). Thank You.

**2**. Why was the sample size computed to detect HBV rather than to estimate with some precision the prevalence of each of the 4 pathogens? A note would be useful as it is too late to amend the sample size anyway

**Response**: Thanks for the comment. In this study, the main or primary population of interest was the HBV patients – in which we are also trying to estimate the prevalences of each of the 4 infections. Also, looking at the prevalence of some of the other pathogens we found among the HBV, for instance, 3% each of *P. falciparum*, and *S. typhi*, it could have required larger sample sizes to make the desired power (besides data unavailability on this in Ghana)– and that would mean more fund and other resource requirements– which are not available. Using the primary HBV infected population has helped to effectively use our (meagre) resources, while achieving the study objectives too. Thank You

3. Table 1 is not well formatted for Epidemiology/Public Health use. Please redo the table to have three columns. On for variable, for absolute counts, and for relative frequency. The second column should add up to 120, whereas the third is 100% per variable

**Response**: Table 1 has been revised as below:

**Table 1: Sociodemographic Characteristics of the Study Participants**

| **Variable** | **Frequency** | **Percentage (%)** |  |
| --- | --- | --- | --- |
| ***Gender***  Male  Female    ***Age (Years)***  ≤ 20  21- 30  31- 40  41 - 50  >50    ***Region of Origin***  Eastern  Greater Accra  Northern  Oti  Ahafo  Volta  Ashanti  Bono East  Bono  Ahafo  Central  Savannah  Upper East  Upper West  Western  Western North  ***Ethnic Group***  Akan  Ewe  Ga-Dangme  Grusi  Guan  Gurma  Bissa  Mole-Dagbani  HFE  Uneducated  Primary  Secondary  Tertiary  ***Marital Status***  Previously Married  Married  Single  ***FM With HBV Positive***  Spouse  Sibling  Not that I know of  None | 75  45  5  62  38  14  1  21  40  4  3  2  4  6  4  1  1  5  7  9  1  4  8  54  9  29  2  2  4  2  18  2  24  67  27  41  65  14  1  3  111  5 | 62.5  37.5  4.2  51.7  31.7  11.7  0.8  17.5  33.4  3.3  2.5  1.7  3.3  5.0  3.3  0.8  0.8  4.2  5.8  7.5  0.8  3.3  6.7  45.0  7.5  24.2  1.7  1.7  3.3  1.6  14.9  1.7  20.0  55.8  22.5  34.2  54.2  11.7    0.8  2.5  92.5  4.2 |  |
|  |  |  |  |

***Table 1****: Sociodemographic Characteristics were presented as frequency (percentage, %). G. Accra: Greater Accra, HFE: Highest Formal Education, FM: Family Member, HBV: Hepatitis B Virus*

Thank You

4. Please fill out the STROBE checklist for the cross-sectional form [https://www.strobe-statement.org/checklists/]. Address any item missing in the manuscript.

**Response**: Strobe checklist completed and attached as **R1 13 STROBE Checklist**

5. Tables 2 and for the continuous variables:

**Comment**: - OK for the mean and Standard Deviation (SD), and independent t-tests. However, the literature on these variables indicates that they are very skewed. Please show the medians and the IQR as well.

- Please, remove the p-values for HBV vs S. typhi and HBV vs P. falciparum. The Ns are too small.

**Response**: Table 2 revised below, and the p-values have been removed

**Table 2a: Effects of Bacteria pathogen (H. pylori and S. typhi)** **coinfections on liver function markers of HBV (HBsAg+) persons**

|  | **Liver Function**  **Markers (LFM’s)** | HBV only (n= 42)  Mean (±SD)  Median (IQR) | HBV -H. Pylori (n = 49)  Mean (±SD)  Median (IQR) | *P-value* |
| --- | --- | --- | --- | --- |
|  | Total Bilirubin (µmol/L)  Direct Bilirubin (µmol/L)  Indirect Bilirubin, calc.  Total Protein (g/L)  Albumin (g/L)  Globulins, calc.  ALT (U/L)  AST (U/L)  γGT (U/L)  ALP (U/L)  Platelets x 10^9^/L  AST/ALT ratio  FIB-4 | 13.5(5.2)  13.5 (10.4 - 16.6)  6.1 (3.2)  5.7 (3.5 - 7.9)  7.3 (4.2)  6.7 (3.55 - 9.85)  71.5 (8.2)  71.7 (67.2 - 76.2)  40.0 (6.3)  38.6 (34.1 - 43.1)  31.5 (7.6)  31.6 (27.65 - 35.55)  23.5 (10.1)  21.0 (15.0 - 27.0)  27.8 (22.1)  26.20 (20.45 - 31.95)  31.7 (13.4)  32.0 (26.0 - 38.0)  161.1 (51.8)  152 (116 - 188)  197.4 (49.5)  190 (155 - 225)  1.35 (1.39)  1.030 (0.785 - 1.275)  0.92 (0.60)  0.85 (0.54 - 1.16) | 15.2 (6.9)  14.6 (10.9 - 18.3)  6.3 (3.0)  6.4 (4.25 - 8.55)  8.9 (5.8)  8.1 (4.7 - 11.5)  71.3 (9.7)  70.9 (65.25 - 76.55)  39.8 (8.0)  38.4 (33.55 - 43.25)  31.5 (8.9)  31.2 (26.5 - 35.9)  *33.5 (15.1)*  31.8 (20.55 - 43.05)  34.8 (18.8)  33.2 (25.2 - 41.2)  29.7 (10.7)  30.0 (23.0 - 37.0)  152.0 (56.0)  144 (105 - 183)  191.9 (54.5)  191 (156 - 226)  1.28 (1.13)  1.000 (0.675 - 1.325)  1.16 (1.12)  0.930 (0.625 - 1.235) | 0.31  0.98  0.23  0.91  0.94  0.96  ***<0.001^**^***  0.07  0.25  0.32  0.44  0.71  0.21 |
|  | | HBV only (n= 42) | HBV -S. typhi (n = 3) |  |
|  | Total Bilirubin (µmol/L)  Direct Bilirubin (μmol/L)  Indirect Bilirubin,calc.  Total Protein (g/L)  Albumin (g/L)  Globulins, calc.  ALT (U/L)  AST (U/L)  γGT (U/L)  ALP (U/L)  Platelets x10^^9^/L  AST/ALT ratio  FIB-4 | 13.5 (5.2)  13.5 (10.4 - 16.6)  6.1 (3.2)  5.7 (3.5 - 7.9)  7.3 (4.2)  6.7 (3.55 - 9.85)  71.5 (8.2)  71.7 (67.2 - 76.2)  40.0 (6.3)  38.6 (34.1 - 43.1)  31.5 (7.6)  31.6 (27.65 - 35.55)  23.5 (10.1)  21.0 (15.0 - 27.0)  27.8 (22.1)  26.20 (20.45 - 31.95)  31.7 (13.4)  32.0 (26.0 - 38.0)  161.1 (51.8)  152 (116 - 188)  197.4 (49.5)  190 (155 - 225)  1.35 (1.39)  1.030 (0.785 - 1.275)  0.92 (0.60)  0.85 (0.54 - 1.16) | 23.6 (8.5)  25.9 (25.9 - 25.9)  12.9 (5.9)  12.1 (12.1 - 12.1)  10.7 (7.6)  8.5 (8.5 - 8.5)  84.2 (5.0)  84.5 (84.5 - 84.5)  *53.2 (2.4)*  54.0 (54.0 - 54.0)  30.8 (6.8)  29.2 (29.2 - 29.2)  43.7 (19.3)  50.0 (50.0 - 50.0)  30.7 (6.7)  29 (29 - 29)  38.7 (27.7)  35 35 (35 - 35)  201 (72.0)  219 (219 - 219)  184.7 (62.3)  167 (167 - 167)  0.83 (0.45)  0.86 (0.86 - 0.86)  0.60 (0.18)  0.69 (0.69 - 0.69) |  |
|  |  |  |  |  |

***Table 2a: Data is presented as mean ± SD. * = p-values< 0.05, ** = p-values< 0.01 were considered statistically significant. ALT – Alanine transaminase, AST – Aspartate transaminase. GGT - Gamma-glutamyl transferase, ALP – Alkaline phosphatase, FIB-4 - fibrosis 4 indices.***

**Table 2b: Effects of Protozoa Parasites (*T. gondii and P. falciparum)* on** **coinfections on liver function markers of HBV (HBsAg+) persons**

|  | **Liver Function**  **Markers (LFM’s)** | HBV only (n= 42)  Mean (±sd) | HBV -T. gondii (n = 23) | *P-value* |
| --- | --- | --- | --- | --- |
|  | Total Bilirubin (μmol/L)  Direct Bilirubin (μmol/L)  Indirect Bilirubin, calc.  Total Protein (g/L)  Albumin (g/l)  Globulins, calc.  ALT (U/L)  AST (U/L)  γGT (U/L)  ALP (U/L)  Platelets x10^^9^/L  AST/ALT ratio  FIB-4 | 13.5(5.2)  13.5 (10.4 - 16.6)  6.1 (3.2)  5.7 (3.5 - 7.9)  7.3 (4.2)  6.7 (3.55 - 9.85)  71.5 (8.2)  71.7 (67.2 - 76.2)  40.0 (6.3)  38.6 (34.1 - 43.1)  31.5 (7.6)  31.6 (27.65 - 35.55)  23.5 (10.1)  21.0 (15.0 - 27.0)  27.8 (22.1)  26.20 (20.45 - 31.95)  31.7 (13.4)  32.0 (26.0 - 38.0)  161.1 (51.8)  152 (116 - 188)  197.4 (49.5)  190 (155 - 225)  1.35 (1.39)  1.030 (0.785 - 1.275)  0.92 (0.60)  0.85 (0.54 - 1.16 | 15.2 (7.2)  14.8 (9.35 - 20.25)  5.8 (2.6)  6.1 (4.15 - 8.05)  9.3 (5.9)  8.2 (4.2 - 12.2)  68.9 (9.4)  70.4 (62.4 - 78.4)  38.2 (6.9)  37.3 (31.45 - 43.15)  31.7 (7.9)  29.7 (25.35 - 34.05)  27.3 (14.6)  23.0 (12.75 - 33.25)  29.0 (12.3)  28.4 (18.8 - 38.0)  29.7 (10.8)  26.5 (20.0 - 33.0)  153 (52)  150 (117 - 183)  203 (50)  215 (177 - 253)  1.24 (0.60)  1.14 (0.79 - 1.49)  0.94 (0.38)  0.96 (0.72 - 1.20) | 0.26  0.76  0.23  0.12  0.24  0.75  0.16  0.83  0.69  0.71  0.54  0.64  0.87 |
|  | | HBV only (n= 42) | HBV -P. falciparum (n =3) |  |
|  | Total Bilirubin (μmol/L)  Direct Bilirubin (μmol/L)  Indirect Bilirubin, calc.  Total Protein (g/L)  Albumin (g/L)  Globulins, calc.  ALT (U/L)  AST (U/L)  γGT (U/L)  ALP (U/L)  Platelets x10^^9^/L  AST/ALT ratio  FIB-4 | 13.5(5.2)  13.5 (10.4 - 16.6)  6.1 (3.2)  5.7 (3.5 - 7.9)  7.3 (4.2)  6.7 (3.55 - 9.85)  71.5 (8.2)  71.7 (67.2 - 76.2)  40.0 (6.3)  38.6 (34.1 - 43.1)  31.5 (7.6)  31.6 (27.65 - 35.55)  23.5 (10.1)  21.0 (15.0 - 27.0)  27.8 (22.1)  26.20 (20.45 - 31.95)  31.7 (13.4)  32.0 (26.0 - 38.0)  161.1 (51.8)  152 (116 - 188)  197.4 (49.5)  190 (155 - 225)  1.35 (1.39)  1.030 (0.785 - 1.275)  0.92 (0.60)  0.85 (0.54 - 1.16 | 26.7 (6.0)  28.4 (28.4 - 28.4)  7.3 (3.4)  6.6 (6.6 - 6.6)  19.4 (5.0)  20.0 (20.0, 20.0  74.3 (15.2)  81.0 (81.0, 81.0)  50.5 (14.5)  50.7 (50.7, 50.7)  23.7 (9.5)  20.9 (20.9 - 20.9  39.3 (15.1)  43.0 (43.0 - 43.0)  36.3 (7.6)  38.0 (38.0 - 38.0)  28.0 (4 .6)  27.0 (27.0 - 27.0)  179.7 (54.7)  162.0 (162.0 - 162.0)  201.7 (50.6)  198 (198.0 - 198.0)  0.99 (0.26)  0.93 (0.93 - 0.93)  0.90 (0.25)  0.85 (0.85 - 0.85) |  |
|  |  |  |  |  |

***Table 2b: Data is presented as mean (± SD). * = p-values< 0.05, ** = p-values< 0.01 were considered statistically significant. ALT – Alanine transaminase, AST – Aspartate transaminase. GGT - Gamma-glutamyl transferase, ALP – Alkaline phosphatase, FIB-4 - fibrosis 4 indices.***

6. Figure 1 and prevalence estimates:

- please add 95% confidence interval

- Was there any HBV patients with multiple pathogens?

- Figure please sort the bars according to the prevalence

**Response**: Please, it looks a bit odd to add CI’s to **these single point prevalence values**, as each have no variability nor uncertainty around them. Can we please leave it out. Otherwise, each will have a CI of 0.0. Thank You.

All HBV patients with multiple pathogens were excluded.

The bars of the figure have been sorted according to the prevalence. Thank You

**Reviewer #1**: Reconsider the title; it should not have non-standard abbreviations. Write-out in full HBV and species names i.e., “Prevalence of Helicobacter pylori, Salmonella typhi, Plasmodium falciparum, and Toxoplasma gondii infections and levels of liver function markers among Hepatitis B Virus infected Ghanaians: A cross-sectional study in the Greater Accra Region”

**Response**: Title of Paper re-considered and revised to read: “Prevalence of *Helicobacter pylori, Salmonella typhi, Plasmodium falciparum*, and *Toxoplasma gondii* infections and levels of liver function markers among Hepatitis B Virus infected Ghanaians: A cross-sectional study in the Greater Accra Region”. Thank You

ABSTRACT

**Comment**: Rephrase for clarity; for example, the first sentence could be rephrased as “Coinfection of humans with Hepatitis B Virus (HBV) and non-viral pathogens may worsen the outcome of HBV infection on the liver.”

**Response**: The abstract has been rephrased for clarity. This includes the first sentence to read “Coinfection of humans with Hepatitis B Virus (HBV) and non-viral pathogens may worsen the outcome of HBV infection on the liver”. Thank You

Comment: Define ‘LFM’s’; ALT, etc., at first use of term

**Response**: Please, abbreviations have been written in full on their first use from the Abstract through to the Discussions. Thank You

**Comments**: You state that H. pylori, S. typhi, P. falciparum, and T. gondii were screened for, from the blood using lateral flow immunochromatographic assays (LFM). Yet, lines 153-155 (Methods section - in the main text) you state that “The P. falciparum positive samples were confirmed by a trained microscopic examination of thin and thick film prepared for blood smear.” Please reconcile.

Response: Please the sentences have revised and reconciled accordingly. Thank You

INTRODUCTION

**Comment**: Lines 70-71: GAR has already been defined but you continue to use the full term (Greater Accra Region) thereafter.

**Response**: Please, full use of Greater Accra Region, has been limited to its first use in the abstract and the body of the manuscript. Thank You

METHODS

**Comment**: Regarding P. falciparum detection, reconcile the information in lines 153-155 with that in lines 41-43 (abstract).

**Response**: P. falciparum detection information has been reconciled (lines 160 -166), abstract (41-44)

Furthermore, immunological/lateral flow-based assays (LFM) were used to detect all the pathogens under study except P. falciparum that was detected by using microscopy. Yet, LFM assays for detection of P. falciparum also do exist, so I wonder what the rationale of using microscopy, which has a very low sensitivity, was.

Response: Despite the low sensitivity of blood film microscopy for the detection of *P. falciparum*, it remains the gold standard *for P. falciparum* diagnosis in Ghana. This study used both the immunochromatographic (lateral flow immunoassay) technique, and the microscopy technique to improve the sensitivity and specificity of the detection outcome. Thank You

**Comments**: Relatedly, for S. typhi, the immunology-based assays need to be interpreted with caution as they usually imply prior exposure, not necessarily recent/active infection and tend to have a very high sensitivity.

**Response**: Comments well noted. We sincerely share your caution on the use of the immunochromatographic technique for the S. typhi detection, however, to minimize the errors associated with the recent and past infections (clarity), we selected test devices which can detect both recent/active infection (demonstrated by presence of typhoid IgM) and late/chronic infection (demonstrated by presence of typhoid IgG) . Thank You.

DISCUSSION

**Comments**: The authors have not discussed the key limitations regarding detection of certain pathogens under study, which have been pointed out in methods.

First, immunological/lateral flow-based assays (LFM) were used to detect all the pathogens under study except P. falciparum that was detected by using microscopy. Yet, LFM assays for P. falciparum detection do exist; I wonder what the rationale of using microscopy, which has a very low sensitivity, was. One is tempted to imagine that the prevalence of P. falciparum could be higher than what was reported if a more sensitive detection method was adopted.

Relatedly, for S. typhi, the immunology-based assays need to be interpreted with caution as they usually imply prior exposure, not necessarily recent/active infection and tend to have a very high sensitivity.

**Response**: We are most grateful to you for such a thorough review. Please, we have explained these concerns in our previous responses. Much Grateful!
