# Supplementary material for: Prevalence of Helicobacter pylori, Salmonella typhi, Plasmodium falciparum, and Toxoplasma gondii infections and levels of liver function markers among Hepatitis B Virus infected Ghanaians: A cross-sectional study in the Greater Accra Region
Source: PLOS Glob Public Health. 2025 Sep 5;5(9):e0005132. doi: 10.1371/journal.pgph.0005132 (PMC12412962; doi:10.1371/journal.pgph.0005132)
Supplement: Attachment — Submitted filename: R2 10 Response to Reviewers.docx [file pgph.0005132.s005.docx]

15^th^ May 2025

Dear Reviewer (s) & Editor,

**Point-by-point response to reviewers (Round 2)**

(**RE**: Prevalence of H. pylori, S. typhi, P. falciparum, and T. gondii infections and levels of liver function markers among HBV infected Ghanaians: A cross-sectional study in the Greater Accra Region) (**PPGPH-D-24-02628R1**)

Thank you for your thorough review, suggestions, and comments. Please find responses:

**Questions/ Comments:**

***Additional Editor Comments:***

It was considered necessary to invite additional reviewers after the previous reviewer declined. One of the reviewers is more positive about your study, but the other reviewer has raised remaining concerns that need to be addressed.

**Response**: Thank You Editor. We will address them please. Thank You

***Reviewer #2:***

This study attempts to provide an overview regarding the prevalence of Heliobacter pylori, Salmonella typhi, Plasmodium falciparum, and Toxoplasma gondii among HBV-infected persons in the Greater Accra Region of Ghana. In addition, the levels of liver function markers were determined in the coinfected patients. The prevalence of H. pylori and T. gondii was high among HBV-infected patients. Liver function tests were elevated in coinfected cases. This study well describes the current status (prevalence) of these infections in HBV-infected patients, and the effects of these coinfections on liver function tests. The findings of this study contribute further evidence for informing reforms to the national guidelines for the management, prevention, and control of these coinfections in HBV positive patients.

**Response**: Thanks for taking the time to review our paper. Many thanks for the summary and comments shared. We appreciate it.

***Reviewer #3:***

This study is to determine the Prevalence of Helicobacter pylori, Salmonella typhi, Plasmodium falciparum, and Toxoplasma gondii infections and levels of liver function markers among Hepatitis B Virus infected Ghanaians. I have some few comments about this study

**Response**: Thanks for taking the time to review this work. Your comments are well acknowledged, and we are hopeful that our responses in this (2^nd^ Review) and that in the 1^st^ Review (previously shared), will adequately address your concerns. Thank You.

1. The authors tested for antibodies to certain infections in asymptomatic patients with viral hepatitis. However, the presence of antibodies does not necessarily indicate a current infection, particularly for H. pylori, which has a high prevalence in Ghana (44-70%). Furthermore, the typhoid antibody test (IgM) has a low specificity of approximately 50%, leading to a high rate of false positive results, especially in asymptomatic patients. Therefore, the authors cannot conclusively determine that these organisms were present in the patients at the time of sampling.

**Response**: These comments are well noted and are largely not in doubt. However, the tests and the devices used all have sensitivity and specificity above 95%, they were all quality controlled with samples from the NMIMR using the ELIZA technique. and were comparable to the gold standards. Also, the testing devices used (including the CTK Biotech device for S. typhi, with a Specificity of over 95%) were the combo type with the ability to detect and discriminate between IgG and IgM – which are for past/chronic and current/acute infections respectively (lines 152 -166). Moreover, results from these Ag/Ab tests form part of the decision to treat and manage these infections in Ghana. Thank You.

2. The authors also found a correlation between high ALT levels and positive H. pylori antibody tests. However, the average ALT levels were within the normal range, suggesting that there was no significant inflammation in these patients. Therefore, it cannot be concluded that H. pylori infection contributes to increased inflammation or accelerates liver disease progression in these patients.

**Response**: Thanks for your comments. Yes, the ALT activities/levels were within the normal reference range – and by this you are (surely) referring to apparently healthy subjects. However, this comparison was between HBV-only infected subjects and HBV-H. Pylori co-infected subjects, and our results (lines 208 to 210, Table 2a), discussions in reference to other studies (lines 259 to 270), and conclusions (330 to 331) acknowledged this statistically significant elevation of ALT (P <0.001), without commenting or concluding on its hepato-inflammatory status – which is a subject of clinical significance (when ALT would have been above the normal reference range), than statistical significance. Thank You.

3. The study's findings have limited implications for the monitoring or management of viral hepatitis B in Ghana. The authors should also note that any form of infection can cause inflammation of the liver but tends to be acute without causing chronic infection

**Response**: Thanks for your comments. These views are well noted and acknowledged. Moreover, to the best of the study’s goal, and within the study’s objectives, methods and limitations, participants with some of these other infections/co-infections were excluded (lines 117 to 120). Your comments are well noted. Thank You.

4. The results would have been more meaningful if the authors had compared patients with these infections and hepatitis B-associated liver cirrhosis or hepatocellular carcinoma to those with hepatitis B without end-stage liver disease, allowing for a more informative analysis of potential correlations and disease progression.

**Response**: Thanks for your comments. We sincerely appreciate these insights; however, the objectives of this (current) study were to find how these infections prevail among HBV infected subjects, and the effect of such co-infections (if present) on liver function markers, compared with those infected with only HBV. These insights would be considered in future, hopefully in the light of our previous study on cirrhosis deaths in Ghana (Agbozo et al., 2022) and the Chronic liver diseases verses HDV study, currently ongoing.

We appreciate your insightful comments and queries and hope our responses address your concerns.

Many Thanks to You, Reviewers.

________________________________________
