# Supplementary material for: Prevalence of Helicobacter pylori, Salmonella typhi, Plasmodium falciparum, and Toxoplasma gondii infections and levels of liver function markers among Hepatitis B Virus infected Ghanaians: A cross-sectional study in the Greater Accra Region
Source: PLOS Glob Public Health. 2025 Sep 5;5(9):e0005132. doi: 10.1371/journal.pgph.0005132 (PMC12412962; doi:10.1371/journal.pgph.0005132)
Supplement: Attachment — Submitted filename: 0 1 R 3 Response to Reviewers.docx [file pgph.0005132.s006.docx]

8^th^ July 2025

Dear Reviewer & Editor,

**Point-by-point response to reviewers**

(**RE**: Prevalence of H. pylori, S. typhi, P. falciparum, and T. gondii infections and levels of liver function markers among HBV infected Ghanaians: A cross-sectional study in the Greater Accra Region) (**PGPH-D-24-02628**)

Thank you for your thorough review, suggestions, and comments. Please find responses:

**Questions/ Comments:**

1. Please review your reference list to ensure that it is complete and correct. If you have cited papers that have been retracted, please include the rationale for doing so in the manuscript text or remove these references and replace them with relevant current references. Any changes to the reference list should be mentioned in the rebuttal letter that accompanies your revised manuscript. If you need to cite a retracted article, indicate the article’s retracted status in the References list and include a citation and full reference for the retraction notice.

**Response**: Please. The references list has been reviewed. It is complete and correct, and none of the citations have been retracted. Thank You.

**2**. Please make sure that the manuscript aligns with the reporting recommendations for cross-sectional studies as found in STROBE: <https://www.strobe-statement.org/> .

**Response**: The STROBE checklist submitted on the first revision (R1 13 STROBE Checklist) has been updated to include the current state of the manuscript (0 R3 STROBE Checklists). Thank You

3. - In the Abstract and Results, please report the 95% confidence intervals for the prevalence estimates for the 4 co-infections

**Response**: The 95% confidence intervals for the prevalence estimates for the 4 co-infections have been reported in the abstract and in the results (Lines 47-49, 206-208). Thank You

4.. - In the Abstract, the statement about elevated markers for S. typhi and P. falciparum co-infected participants is misleading, as the differences are not statistically significant and the number of S. typhi and P. falciparum infections are very small. Given the lack of statistical evidence of differences, please remove these results from the abstract and make sure that the Results and Discussion reflect lack of statistically significant differences for these comparisons.

**Response**: The statistically significant reports on LFMs in HBV-S.typhi and HBV-P.falciparum coinfections have been removed from the abstract, and the results and discussions have been reviewed and updated (Lines 49 -51, 211-215, 236-240, 276-280). Thank You

5. - Please add a clear sentence describing the objective of the study in the last paragraph of the Introduction. Currently, it states "Hence this study".

**Response**: The objective of the study has been clearly written (Lines 105-107) Thank You

We sincerely appreciate your time to review this work. Your comments are well acknowledged, and we hope we have addressed them satisfactorily. Thank You.

**Reviewer #2**

6. This study attempts to provide an overview regarding the prevalence of Heliobacter pylori, Salmonella typhi, Plasmodium falciparum, and Toxoplasma gondii among HBV-infected persons in the Greater Accra Region of Ghana. In addition, the levels of liver function markers were determined in the coinfected patients. The prevalence of H. pylori and T. gondii was high among HBV-infected patients. Liver function tests were elevated in coinfected cases. This study well describes the current status (prevalence) of these infections in HBV-infected patients, and the effects of these coinfections on liver function tests. The findings of this study contribute further evidence for informing reforms to the national guidelines for the management, prevention, and control of these coinfections in HBV-positive patients.

**Response**: Thanks for your comments. We sincerely appreciate.

**Reviewer #3**

The authors response well noted.

I still belief that using antibodies of the co-infections investigated such as the H. pylori antibodies does not indicate current infection especially in endemic countries like Ghana and currently not useful to diagnosed or to confirm eradication rate. Though previous studies show an association of H. pylori infection and HCC in patients with risk factors of HCC, using blood samples to determine H. pylori Ab irrespective of the specificity and sensitivity does not confirm the presence of H. pylori.

**Response**: Reviewer comments are well noted. Thank You

One of the current recommendations for managing hepatitis B is persistently high ALT, that is ALT above the reference range. Though there was a statistically significant association between ALT and H. pylori, the average ALT for those with H. pylori, was it above the reference range of the laboratory values used?

**Response**: No, the average ALT for those with H. pylori was within the reference range of the laboratory values used. Thank You

Many Thanks for your time to review this work. Your comments are well acknowledged, and we hope we have addressed them satisfactorily.

Thank You.

Sincerely,

Eric NY Nyarko,

Corresponding Author
